# Achieving Functional and Aesthetic Harmony Through a Fully Digital Workflow for Lateral Agenesis Rehabilitation: A 12‐Month Follow‐Up

**DOI:** 10.1111/jerd.70004

**Published:** 2025-07-21

**Authors:** Cassiana Koch Scotti, Martha Beteghelli Michielin, Ernesto Benalcázar‐Jalkh, Nathalia Cristina Bortolozzo, Juliana Fraga Soares Bombonatti, Marilia Mattar de Amoêdo Campos Velo

**Affiliations:** ^1^ Department of Operative Dentistry, Endodontics and Dental Materials Bauru School of Dentistry, University of São Paulo‐USP Bauru Brazil; ^2^ Department of Restorative Dentistry School of Dentistry, Sao Paulo State University‐UNESP Araraquara São Paulo Brazil

**Keywords:** aesthetic dentistry, dental veneers, digital flow, digital scanning, facial aesthetics

## Abstract

**Objective:**

Digital technologies are revolutionizing dentistry by introducing advanced methods that enhance treatment planning and execution. In complex cases, these innovations improve communication among interdisciplinary teams and streamline workflows for more personalized and efficient care. This case report demonstrates the aesthetic and functional rehabilitation of a patient with agenesis of lateral incisors, smile asymmetry, and facial skeletal discrepancies, using an integrated digital approach.

**Clinical Considerations:**

The patient presented with disproportionate tooth dimensions, color mismatches, and skeletal issues. Initially, orthognathic surgery was considered to address the mandibular discrepancy, but the patient preferred a conservative treatment plan, which included periodontal regularization, teeth whitening, and the fabrication of lithium disilicate laminate veneers through a fully digital workflow. Additionally, facial aesthetics were enhanced with hyaluronic acid lip augmentation.

**Conclusions:**

This case exemplifies the efficacy of a modern, interdisciplinary strategy in aesthetic dentistry, merging cutting‐edge materials, digital planning, and innovative techniques to tackle both functional and aesthetic concerns. The individualized treatment approach and focus on patient‐specific needs were pivotal in achieving a successful and lasting outcome.

## 1 Introduction

While advancements in dental materials can lead to high success rates, understanding restorative principles is essential to achieve both functional and aesthetic outcomes. In complex cases, a multidisciplinary approach is needed for diagnosis and treatment, starting with a systematic analysis that progresses from facial and dentofacial assessments to dentogingival and dental evaluations. The primary aim is to achieve optimal dentolabial relations that are in harmony with the shape of the teeth and the overall facial appearance [1, 2].

Digital technologies have significantly contributed to the introduction of several innovative techniques, transforming the dental practice. These advances have proven particularly advantageous in complex clinical cases, as digital techniques facilitate communication between professionals and patients, while also fostering an interdisciplinary approach [3]. Clinical studies in dentistry increasingly demonstrate the benefits of integrating digital technologies, particularly in the management of complex cases that require individualized treatment planning and multidisciplinary collaboration. Digital planning combined with 3D printing, for example, has shown superior clinical outcomes compared to traditional methods, and a reduction in the number of required follow‐up appointments [4]. Recent innovations, including CAD/CAM systems and 3D printing [5], digital smile design, artificial intelligence, and intraoral scanning, have significantly enhanced diagnostic precision, treatment predictability, and overall patient experience. These technologies allow clinicians to simulate outcomes, improve communication across specialties, and streamline clinical workflows [6]. Clinical evidence supports these advances. One study comparing traditional and digital crown extension guides in anterior aesthetic restorations found that digital approaches led to shorter surgical and planning times, fewer follow‐ups, and better aesthetic results in terms of gingival architecture, tooth shape, and color.

Similarly, a systematic review on the use of intraoral scanners for digital implant impressions confirmed that, despite some technique‐related variability, modern systems offer clinically acceptable accuracy and are increasingly viable alternatives to conventional impressions [7]. In fixed prosthodontics, a 12‐month clinical trial comparing inlay and onlay restorations fabricated using digital versus conventional impressions found that digital impressions provide reliable marginal adaptation and clinical performance over time, reinforcing their value in both routine and complex restorative cases [7, 8].

The expanding body of clinical evidence highlights the transformative impact of digital technologies on dental practice, demonstrating their capacity to enhance procedural efficiency, improve precision in treatment execution, and support more personalized patient care, particularly in complex and multidisciplinary clinical contexts. This evolution in dentistry reflects a broader trend observed across the entire healthcare sector. Recent global developments, especially during the COVID‐19 pandemic, have significantly accelerated the integration of digital tools in clinical care and research [8]. The widespread adoption of telehealth, remote patient monitoring, and digital platforms for clinical trial management has reshaped how healthcare is delivered and studied. Technologies such as artificial intelligence, mobile health applications, and real‐time data tracking are now playing central roles in diagnostics, treatment planning, and clinical decision‐making. These advances, while initially driven by necessity, have established lasting changes in clinical workflows and continue to influence how digital solutions are incorporated into dental practice [8].

Although digital tools improve precision and efficiency in dentistry, clinicians must still have a strong grasp of aesthetic design principles to ensure the final results harmonize with the patient's facial features and expectations. Achieving a balanced smile extends beyond dental procedures, especially in patients with craniofacial discrepancies, such as Class III malocclusion, which can cause facial disharmony. In these cases, it is crucial for clinicians to have a deep understanding of both dental and facial aesthetics, often combining facial harmonization techniques to create a balance between the teeth and facial structure. The successful application of digital workflows in restorative dentistry requires ongoing education and training to effectively manage the complexities of patient‐specific needs and anatomical variations [9]. A comprehensive understanding of both digital technology and orofacial anatomy will ultimately lead to improved clinical results and patient satisfaction in complex restorative cases [10].

Thus, this clinical case report describes the aesthetic and functional rehabilitation of a smile using a fully digital workflow for a patient with missing lateral incisors, smile disharmony, and skeletal facial alterations. By applying advanced digital tools, ceramic laminate veneers, and hyaluronic acid for facial harmonization, we aimed to create a balanced and visually appealing smile that complemented the patient's facial structure, enhancing both function and aesthetics.

## 2 Case Report

A 24‐year‐old female patient presented to the Dental School of Bauru, University of São Paulo (FOB‐USP), reporting significant dissatisfaction with her dental and facial aesthetics. The patient expressed concerns regarding the disproportion in width, color, and height of her teeth, as well as facial skeletal discrepancies. She voluntarily signed an informed consent form authorizing the use of clinical and photographic records for academic and scientific purposes, after receiving detailed information about the procedures involved. This case was conducted in accordance with the ethical standards of the institution and applicable regulatory guidelines.

**FIGURE 1:**
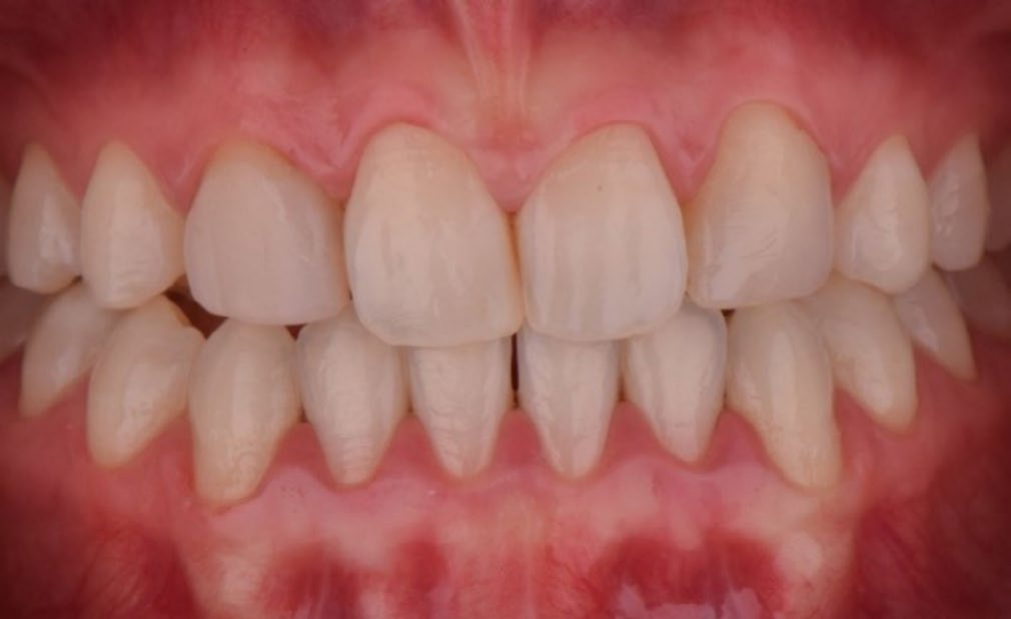
Initial smile of the patient, presenting congenital agenesis of the maxillary lateral incisors (teeth 12 and 22).

**FIGURE 2:**
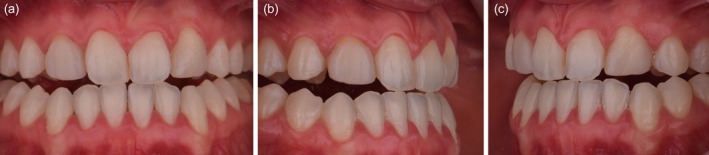
(a) Patient in protrusion, disocclude the posterior teeth, with the exception of the left premolar (element 24); (b) right lateral excursive movement without canine guidance as disocclusion; and (c) left lateral excursive movement without canine guidance as disocclusion.

**FIGURE 3:**
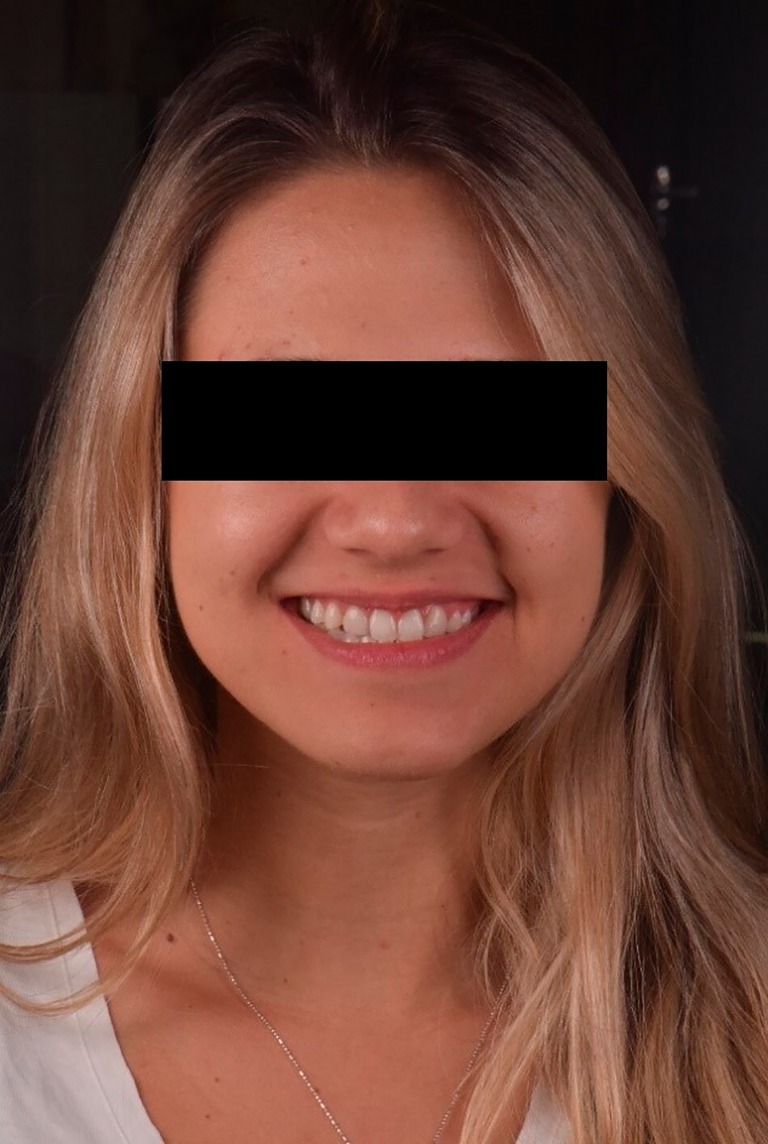
Front view of the patient with excessive mandibular growth.

A comprehensive anamnesis indicated that the patient had a history of 10 years of orthodontic treatment and presented with congenital agenesis of the maxillary lateral incisors (teeth 12 and 22) (Figure 1). The clinical examination revealed large, unsatisfactory resin composite veneers on teeth 13 and 23. The canines (teeth 13 and 23) had been reshaped to function as lateral incisors. Initial clinical examination and photographic documentation were completed; the photographic protocol was carried out with photographs of the smile, of the patient in protrusion (Figure 2a) and in laterality (Figure 2b,c). Figure 3 shows an excessive mandibular growth, and the primary proposed therapy was a surgical orthodontic‐orthognathic procedure to correct the alignment of the bone structures and achieve a skeletal class I, which was discarded by the patient. Different treatment plans were proposed, and a conservative approach was chosen, with periodontal levels regularization and preparation of four lithium disilicate‐based laminate veneers (elements 11,13, 14, 21, 23, and 24).

**FIGURE 4:**
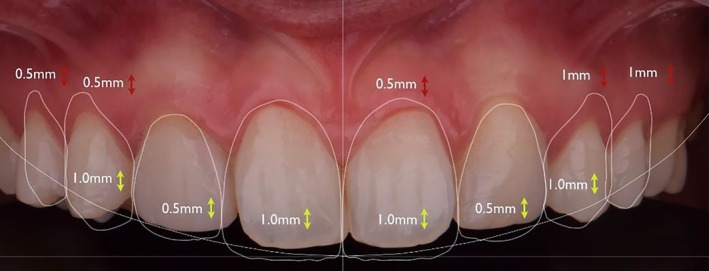
Digital smile planning.

**FIGURE 5:**
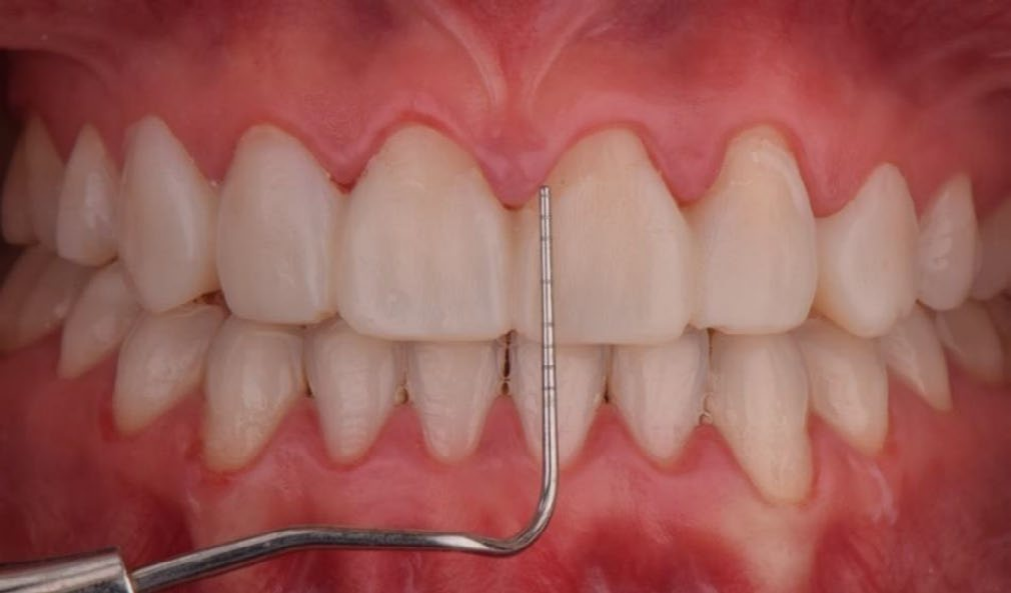
Mock‐up for patient approval and treatment planning.

A digital scan of the patient was performed and the DSD (Digital Smile Design) software was used for aesthetic planning (Figure 4), indicating the need for crown lengthening. Following the completion of the digital plan, the model was printed and the mock‐up was made, which was approved by the patient (Figure 5). A gingivoplasty with an external bevel incision was conducted, removing the gingival tissue in both height and thickness, followed by a 2‐month healing period for gingival margin stabilization. Posteriorly, a dental whitening session was performed using 35% hydrogen peroxide gel (Lase Peroxide, DMC) and hybrid light (LED/LASER, Whitening LASE II, DMC), using a protocol of 3‐min activations, each followed by a 1‐min pause, in a total of two gel exchanges. The procedure was finished with tooth polishing, and a 14‐day period was observed for color stabilization.

**FIGURE 6:**
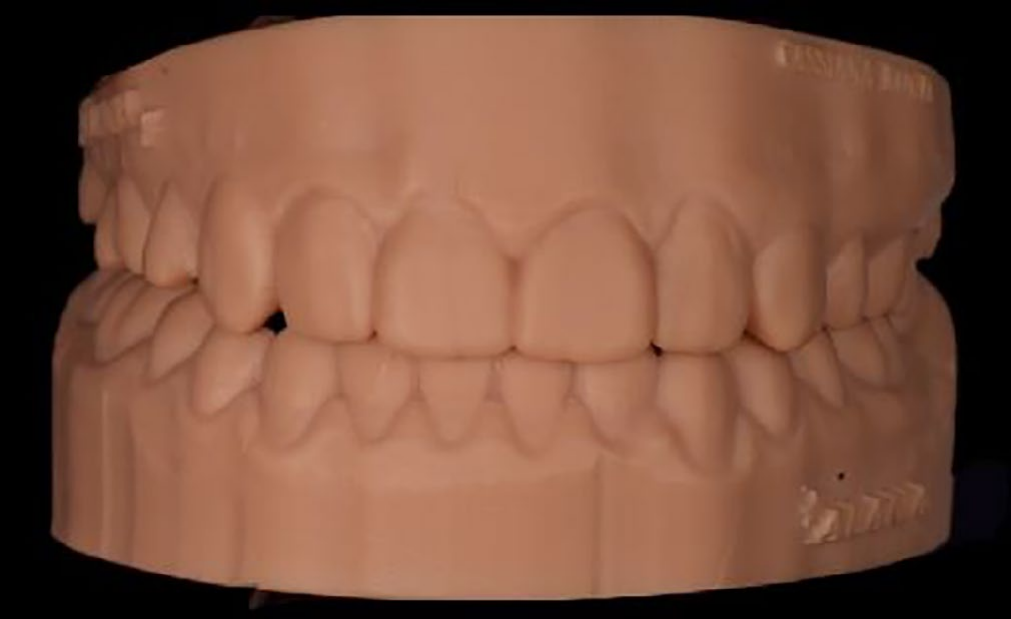
3D‐printed digital model.

**FIGURE 7:**
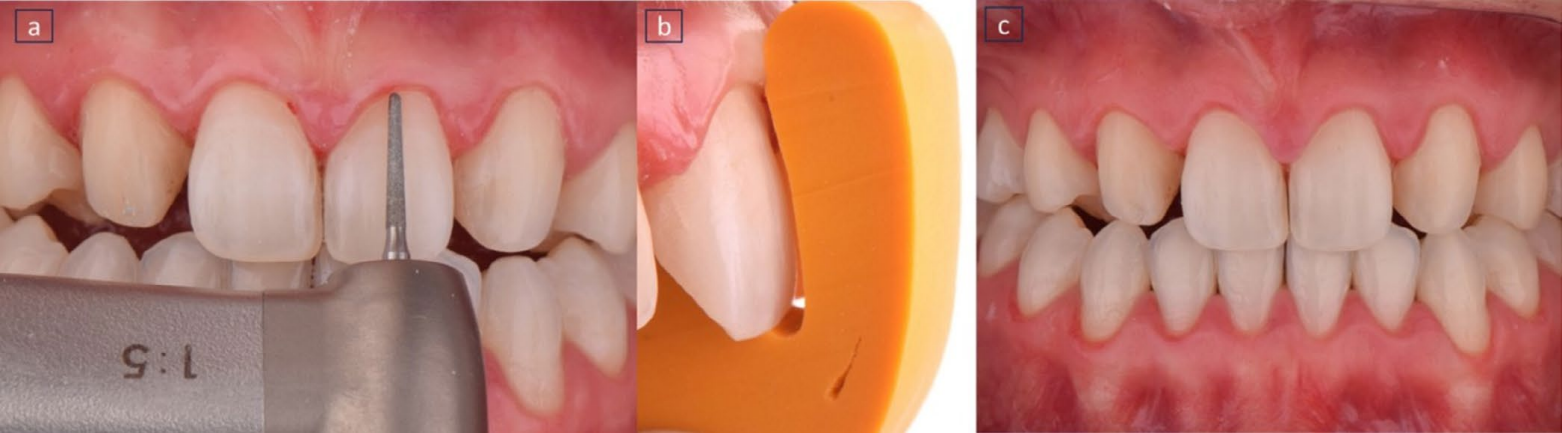
Guides to ensure precise tooth reshaping during preparation.

**FIGURE 8:**
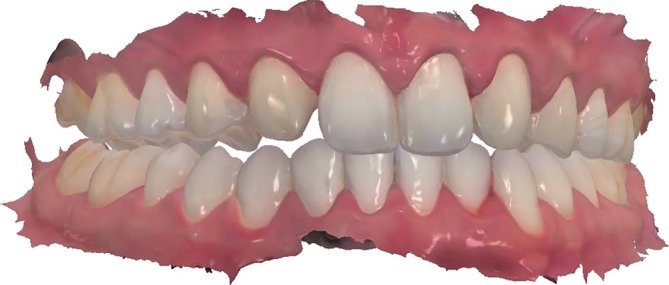
Digital scanning of teeth prepared for the production of ultra‐thin ceramic pieces.

In the treatment sequence, a new digital scan was performed for creating a new digital model. After scanning, a 3D digital model was printed (Figure 6) and used to fabricate a mock‐up, which was used as a guide. Dental preparations were conducted using reduction guides to ensure precise tooth reshaping (Figure 7). These guides help control the amount of tooth structure removed (Figure 7a,b), ensuring optimal space for the final restorations (Figure 7c). The angles were rounded with abrasive discs (FGM) and abrasive rubbers (Enhance‐3M) in order to regularize and avoid retentive areas. Once the teeth were prepared, the veneer shade was chosen, and a new digital scan was sent to the laboratory (Figure 8). The veneers were crafted from lithium disilicate (Ivoclar, CAD/CAM) using the cutback technique, which was employed to improve the aesthetic result.

**FIGURE 9:**
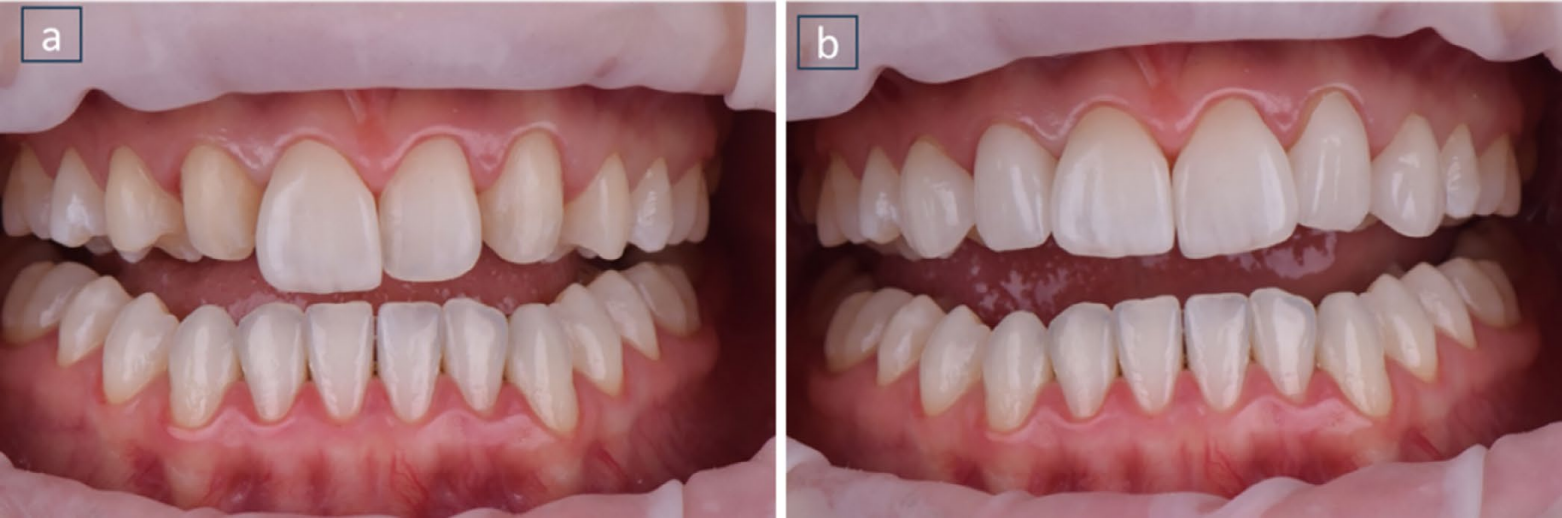
Dry test to check the insertion axis, adaptation, shape, and alignment of all the pieces.

**FIGURE 10:**
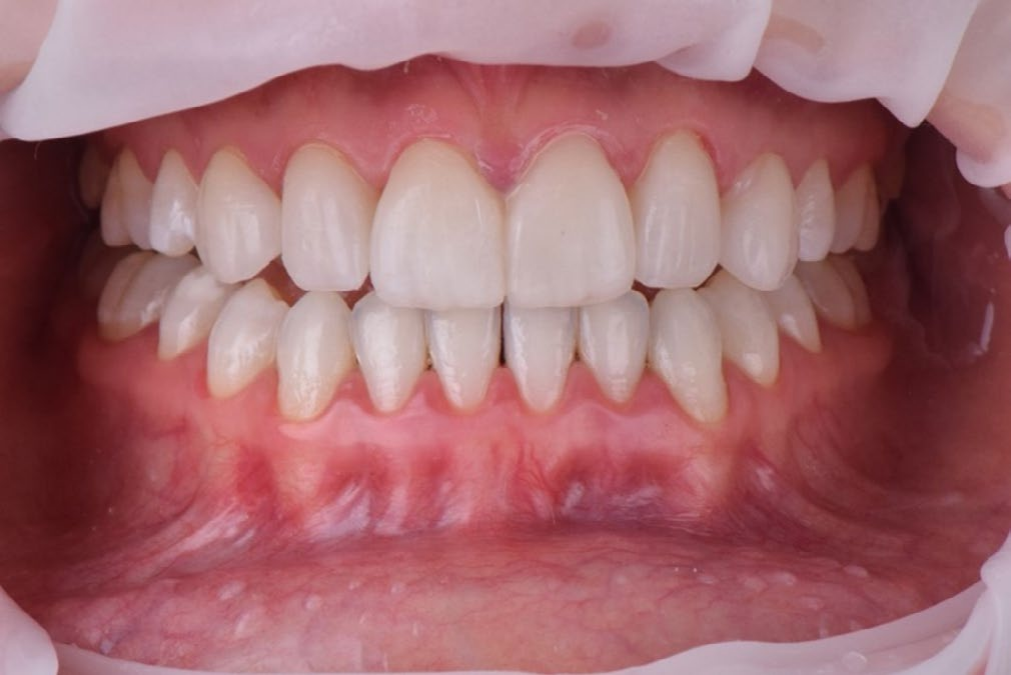
Wet test using a glycerin‐based test paste to finalize the color selection for the cement.

**FIGURE 11:**
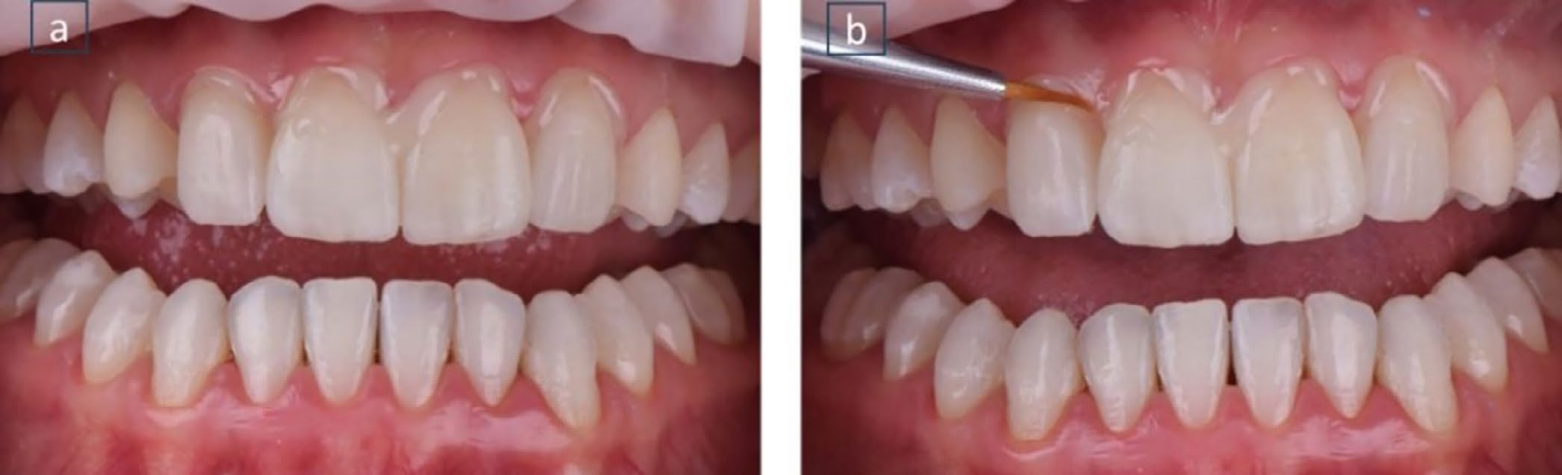
(a) Cementation procedures using a neutral shade; (b) Removal of excess with a brush.

**FIGURE 12:**
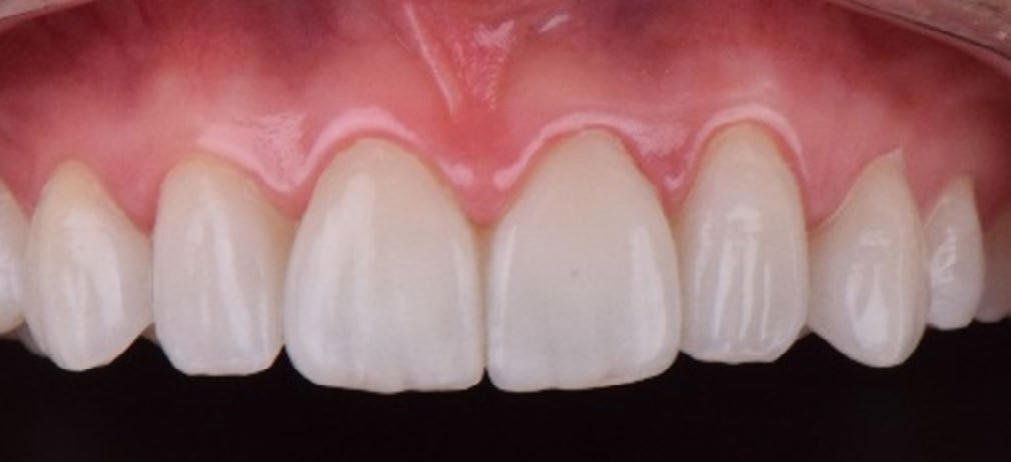
Ceramic laminates fixed immediately after cementation procedure.

**FIGURE 13:**
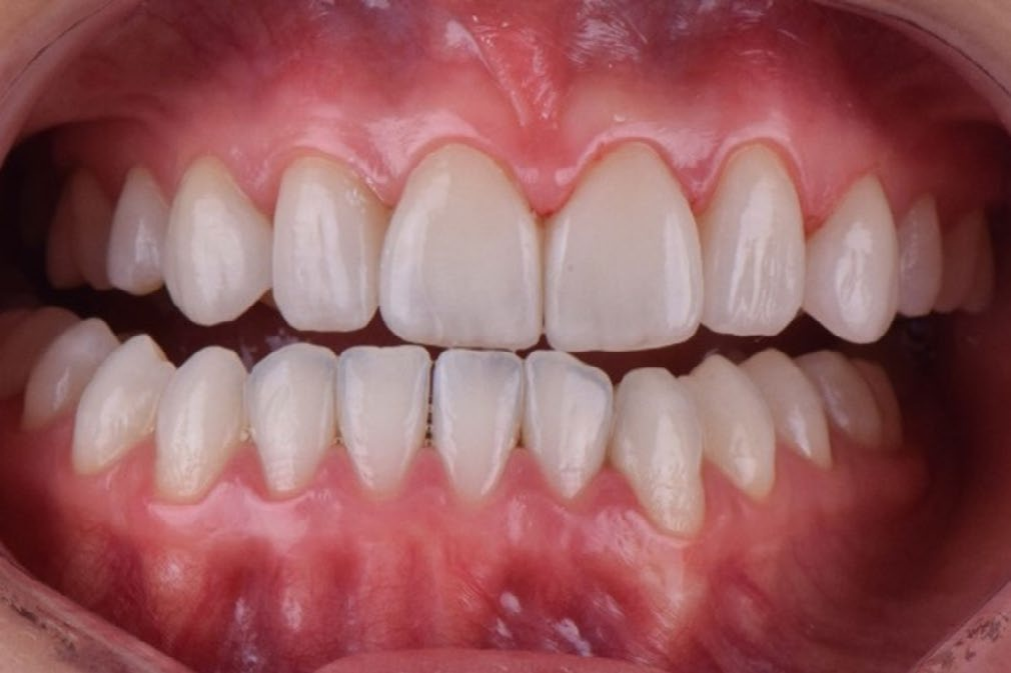
Protrusion movement with disocclusion of posterior teeth.

**FIGURE 14:**
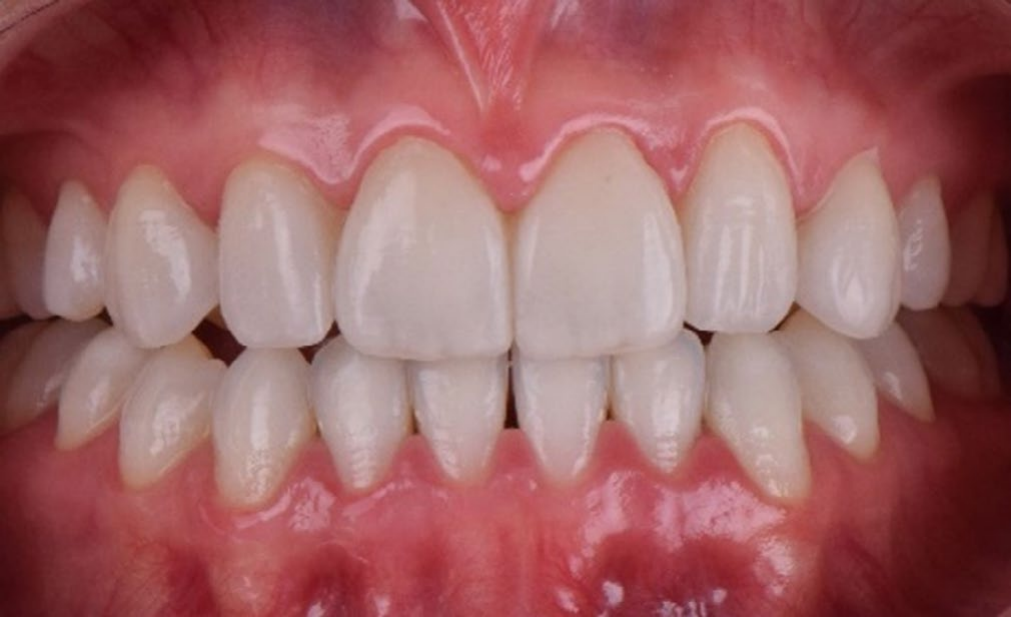
Final smile aspect.

**FIGURE 15:**
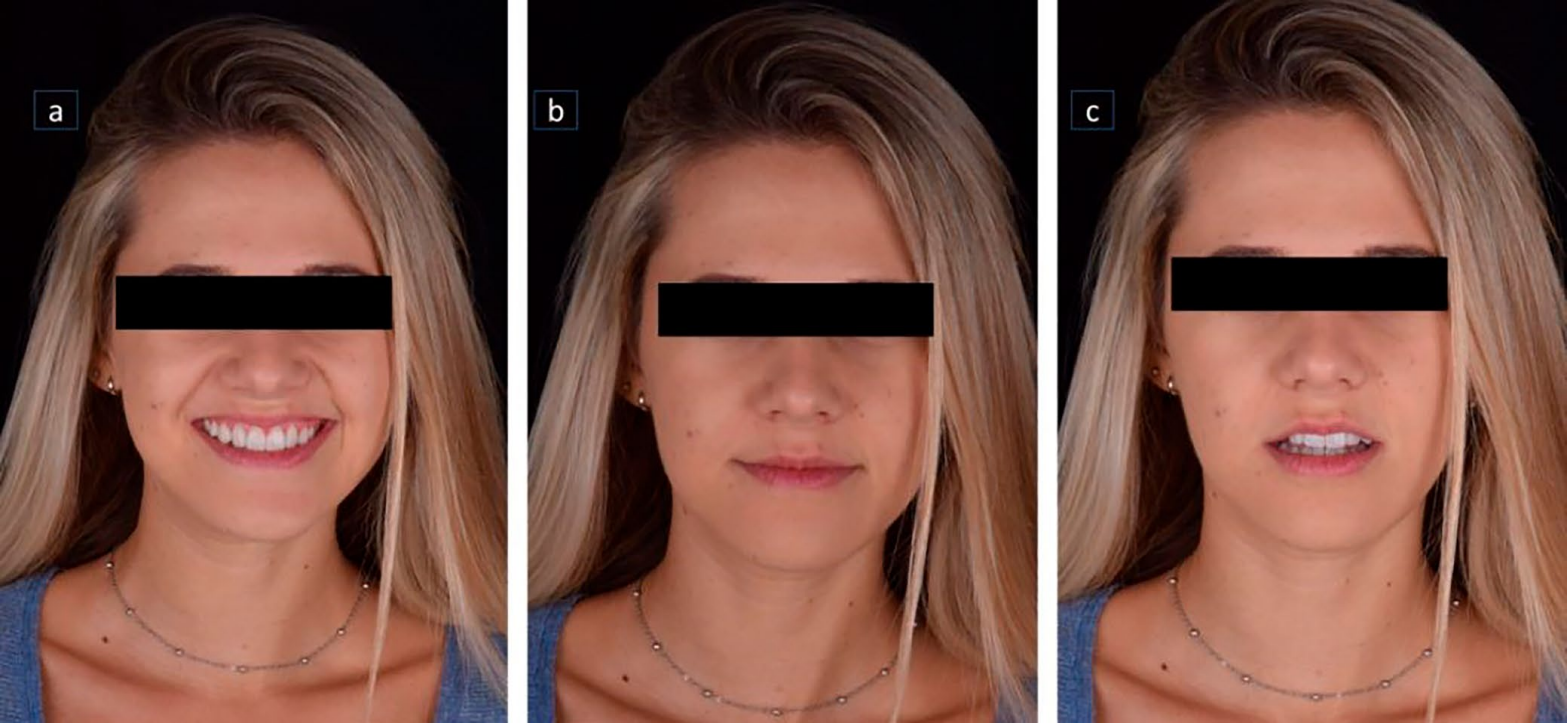
Final smile of the patient, displaying a harmonious smile that aligns with facial aesthetics. (a) Frontal view of the patient smiling; (b) patient with closed lips; and (c) patient with slightly open lips.

For the cementation of the laminate veneers, a dry fit test was performed to check the insertion axis, adaptation, shape, and alignment of all the pieces (Figure 9a,b). Then, a wet test was conducted using a glycerin‐based test paste to finalize the color selection for the cement (AllCem Veneer—Try‐in; FGM Products, Santa Catarina, Brazil) (Figure 10). Prior to the actual cementation, tooth preparation involved etching with 37% phosphoric acid and silane application. The veneer was conditioned with 5% hydrofluoric acid and cemented using Variolink Esthetic in a neutral shade (Figure 11a) and the excess of cement was removed (Figures 11b). The ceramic laminates were fixed immediately after cementation procedure (Figure 12). Protrusion guidance were checked for proper adjustment (Figure 13) and a pleasurable final smile aspect was obtained (Figures 14 and 15a–c).

**FIGURE 16:**
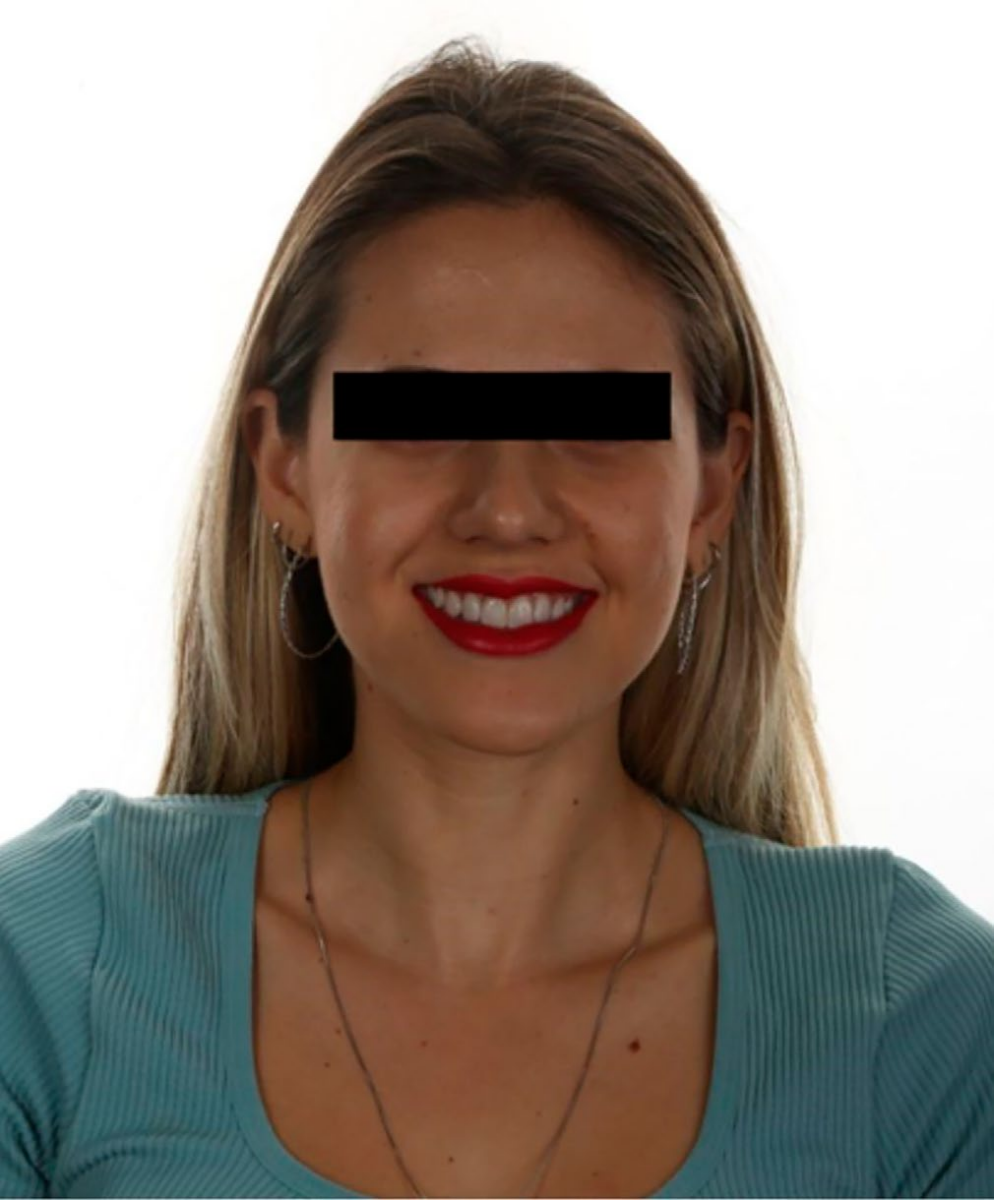
Final smile of the patient after lip filling, providing greater harmony.

**FIGURE 17:**
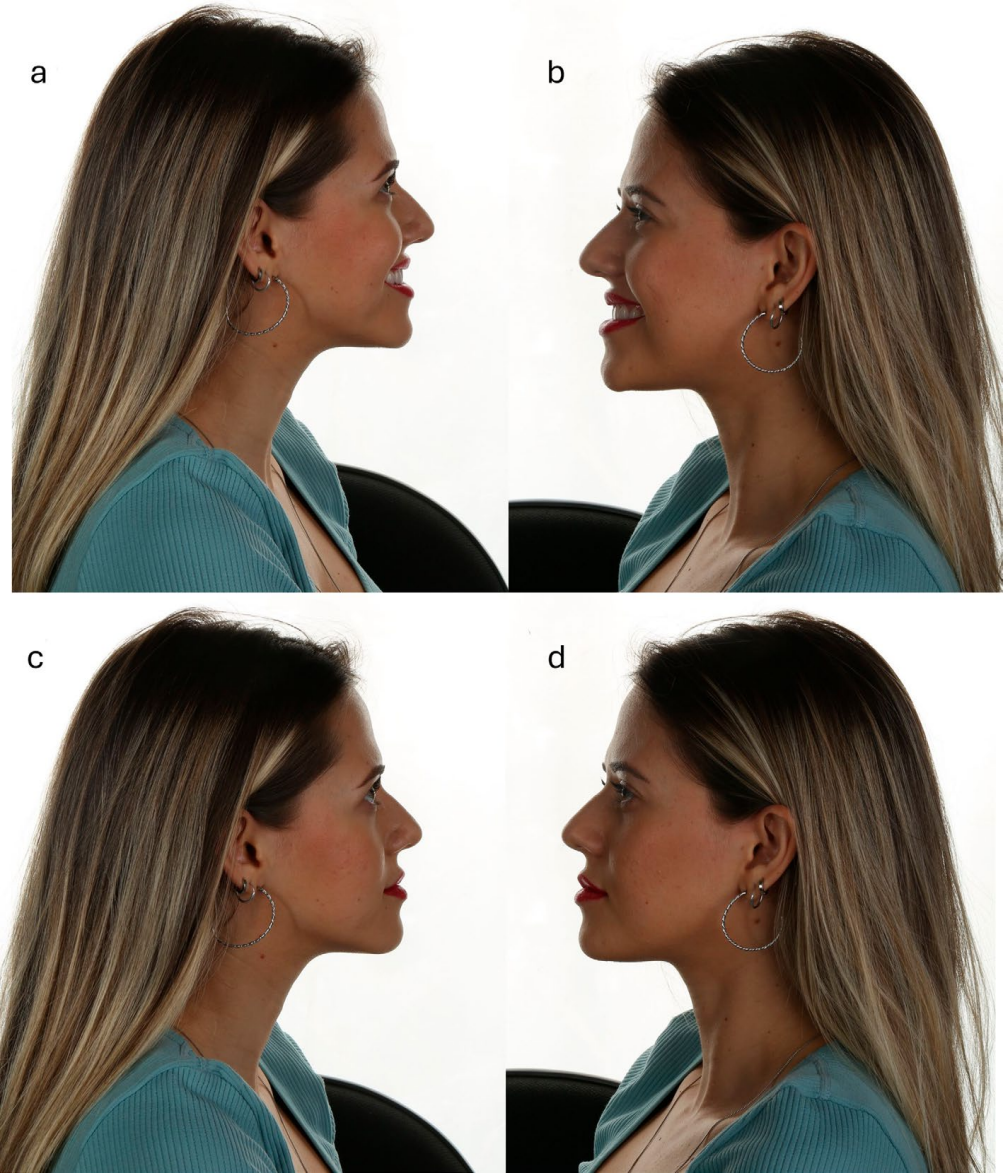
Final facial profile views: (a) right profile smiling, (b) left profile smiling, (c) right profile with lips closed, and (d) left profile with lips closed.

**FIGURE 18:**
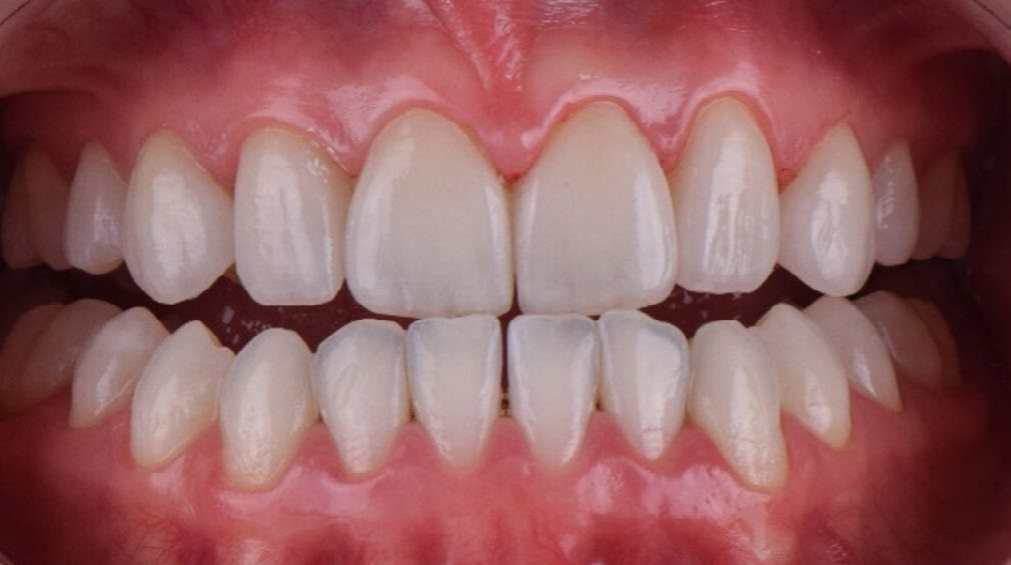
One‐year follow‐up—mandibular protrusion movement.

**FIGURE 19:**
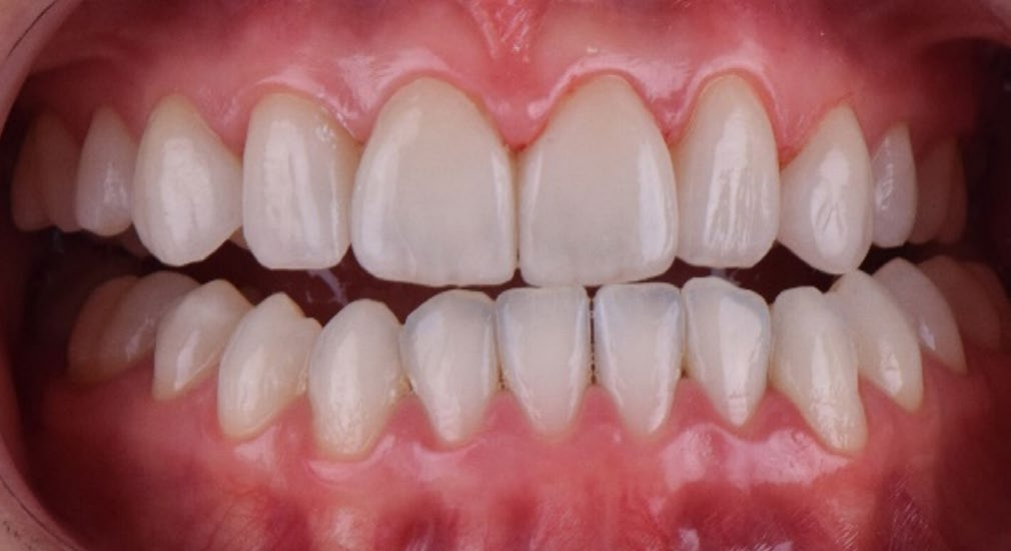
One‐year follow‐up—mandibular lateral movement.

After 12‐month follow‐up, a facial harmonization was performed to enhance the overall aesthetic balance of the patient's face. A lip filling was performed with 1 mL of hyaluronic acid (Juvederm Ultra XC, Allergan). Aiming to deliver a more harmonious proportion between the upper and lower lips, the golden ratio compass was used, maintaining a final proportion of 1:1.618 between the upper and lower lips, respectively [11]. After completing the treatment, follow‐up and maintenance of the veneers were performed to ensure the longevity of the results (Figure 16). Figure 17 illustrates the final facial profile outcomes. The images demonstrate a balanced and harmonious facial profile, reflecting the aesthetic improvements achieved through the multidisciplinary treatment approach. Figures 18 and 19 represent 1‐year follow‐up of mandibular protrusion and right mandibular lateral movement.

## 3 Discussion

The clinical case involves a patient with congenital agenesis of the maxillary lateral incisors (teeth 12 and 22) and a 10‐year history of orthodontic treatment. This condition, common in skeletal class III malocclusion with a prevalence of 12.5% [12], typically requires a multidisciplinary approach, including orthodontics, prosthodontics, and restorative dentistry to restore aesthetics and function [13].

Although the approach related herein does not fully address the underlying skeletal class III malocclusion, it reflects current trends in dental practice emphasizing minimally invasive techniques when appropriate and desired by the patient. Techniques like ultra‐thin veneers can achieve favorable aesthetic outcomes, especially when integrated with digital workflows. Intraoral scanning offers a quick, comfortable, and interactive method for capturing detailed impressions, allowing for precise planning and visualization of dental issues. This approach not only enhances aesthetic results but also minimizes the margin of error typically seen in manual methods [14].

The aesthetic veneer workflow involves several key stages and it has been shown that tooth preparation is more conservative when using a diagnostic mock‐up compared to free‐hand preparation [15]. Digital technologies offer a valuable tool for obtaining aesthetic insights during diagnosis and treatment, a goal that is increasingly achievable today. Furthermore, precise transmission of information to the dental laboratory is essential for the success of restorative procedures [15]. The digital methodology ensures greater standardization in planning, increasing predictability of both functional and aesthetic outcomes, and reducing the number of clinical sessions required [15]. However, challenges such as the high initial cost of equipment and the learning curve for new technologies must be considered [15]. While digital workflows are effective for simple and moderate aesthetic cases, more complex rehabilitations may still benefit from a hybrid approach combining both digital and analog methods.

In this study, gingival levels were adjusted, and the canines (teeth 13 and 23) were reshaped to function as lateral incisors, a common technique for compensating lateral incisor agenesis. However, this approach can lead to aesthetic concerns due to differences in size, shape, and color between the canines and natural lateral incisors. Moreover, the lack of canine guidance and the absence of posterior teeth disocclusion during protrusion can cause discomfort or damage to the dental arches and temporomandibular joint. Canine guidance is essential for disoccluding posterior teeth during lateral movements, reducing masticatory muscle activity and minimizing friction [16].

The decision to fabricate ceramic laminate veneers on the canines and central incisors (teeth 11, 13, 21, and 23) was key to achieving a balanced smile, especially for patients with small, missing, discolored, or misshapen teeth. The use of the cutback technique allowed for high customization, incorporating natural textures and layers of translucent ceramic to enhance the aesthetic outcome [17]. This technique, involving the controlled removal of part of the ceramic after pressing, results in a more refined finish and superior aesthetics, providing a well‐controlled marginal fit and a more realistic appearance [17].

Lip augmentation with hyaluronic acid is widely recognized for its safety and effectiveness. When appropriately indicated, it can contribute to both aesthetic and functional improvements, especially in cases of mandibular discrepancies. Though not the first choice for correcting such issues, when used alongside dental veneers and integrated into a comprehensive treatment plan, these procedures can offer significant benefits. The use of the golden ratio in lip augmentation is a proven strategy for achieving aesthetically pleasing and harmonious results, respecting the patient's unique anatomical features [18].

In conclusion, this case emphasizes the value of a personalized, patient‐centered approach in managing complex dental cases involving congenital anomalies. By integrating advanced materials like lithium disilicate with modern techniques such as digital scanning and the cutback method, the dental team successfully achieved both aesthetic and functional results, overcoming the challenges of the patient's skeletal discrepancies and prior orthodontic treatments. This approach reflects current trends in dentistry, where digital technologies—such as intraoral scanning, diagnostic mock‐ups, and digital smile design—are transforming treatment planning. These innovations allow for more precise, predictable, and customized solutions, aligning with the broader shift in healthcare toward minimally invasive, patient‐focused care. The adoption of digital workflows and advanced materials continues to drive the evolution of restorative dentistry, ensuring enhanced outcomes with greater efficiency and accuracy. Ongoing follow‐up will be crucial to monitor the long‐term success of the restorations and ensure continued patient satisfaction. We also gratefully acknowledge the support of CAPES – Finance Code 001 – for providing open access funding.

## Conflicts of Interest

The authors declare no conflicts of interest.

## Data Availability

Research data not shared.

## References

[1] A. Y. Furuse , E. J. Franco , and J. Mondelli , “Esthetic and Functional Restoration for an Anterior Open Occlusal Relationship With Multiple Diastemata: A Multidisciplinary Approach,” Journal of Prosthetic Dentistry 99, no. 2 (2008): 91–94. 18262008 10.1016/S0022-3913(08)60023-2

[2] L. F. Da Cunha , L. O. Pedroche , C. C. Gonzaga , and A. Y. Furuse , “Esthetic, Occlusal, and Periodontal Rehabilitation of Anterior Teeth With Minimum Thickness Porcelain Laminate Veneers,” Journal of Prosthetic Dentistry 112, no. 6 (2014): 1315–1318. 25156092 10.1016/j.prosdent.2014.05.028

[3] J. Schmalzl , I. Róth , J. Borbély , P. Hermann , and B. Vecsei , “The Impact of Software Updates on Accuracy of Intraoral Scanners,” BMC Oral Health 23 (2023): 219. 37061664 10.1186/s12903-023-02926-y PMC10105929

[4] J. Liu , M. Maihemaiti , L. Ren , et al., “A Comparative Study of the Use of Digital Technology in the Anterior Smile Experience,” BMC Oral Health 24, no. 1 (2024): 492, 10.1186/s12903-024-042285. 38664749 PMC11046787

[5] P. Suksuphan , N. Krajangta , P. P. Didron , T. Wasanapiarnpong , and T. Rakmanee , “Marginal Adaptation and Fracture Resistance of Milled and 3D‐Printed CAD/CAM Hybrid Dental Crown Materials With Various Occlusal Thicknesses,” Journal of Prosthodontic Research 68, no. 2 (2024): 326–335, 10.2186/jpr.JPR_D_23_00089. 37438119

[6] J. P. M. Tribst , G. K. R. Pereira , and C. J. Kleverlaan , “Advancements in Dental Care: The Evolving Landscape of Prosthetic Dentistry,” Journal of Clinical Medicine 13, no. 5 (2024): 1225. 38592049 10.3390/jcm13051225 PMC10932426

[7] A. Schmidt , B. Wöstmann , and M. A. Schlenz , “Accuracy of Digital Implant Impressions in Clinical Studies: A Systematic Review,” Clinical Oral Implants Research 33, no. 6 (2022): 573–585, 10.1111/clr.13951. 35527511

[8] X. Fang and M. Liu , “How Does the Digital Transformation Drive Digital Technology Innovation of Enterprises? Evidence From Enterprise's Digital Patents,” Technological Forecasting and Social Change 204 (2024): 123428, 10.1016/j.techfore.2024.123428.

[9] T. A. Sulaiman , “Materials in Digital Dentistry—A Review,” Journal of Esthetic and Restorative Dentistry 32, no. 2 (2020): 171–181. 31943720 10.1111/jerd.12566

[10] D. M. Sarver , “Orthodontics as an Adjunct to Aesthetic Dentistry,” Journal of Esthetic and Restorative Dentistry 30, no. 1 (2018): 57–68.

[11] H. Galadari and S. H. Weinkle , “Injection Techniques for Midface Volumization Using Soft Tissue Hyaluronic Acid Fillers Designed for Dynamic Facial Movement,” Journal of Cosmetic Dermatology 21, no. 3 (2022): 924–932. 34964234 10.1111/jocd.14700 PMC9303613

[12] C. Swarnalatha , U. Paruchuri , J. S. Babu , et al., “Prevalence of Congenitally Missing Upper Lateral Incisors in an Orthodontic Adolescent Population,” Journal of Orthodontic Science 9 (2020): 15. 33354541 10.4103/jos.JOS_28_19 PMC7749456

[13] T. B. Cozer , M. C. Espaladori , R. M. Alves e Silva , et al., “Faciometrics: A Practical Guide for Orofacial Harmonization,” Mathews Journal of Dermatology 4, no. 1 (2020): 14.

[14] W. M. Ahmed , A. A. Azhari , L. Sedayo , et al., “Mapping the Landscape of the Digital Workflow of Esthetic Veneers From Design to Cementation: A Systematic Review,” Dental Journal 12 (2024): 28. 10.3390/dj12020028 PMC10888163 38392232

[15] A. Lo Giudice , L. Ortensi , M. Farronato , A. Lucchese , E. Lo Castro , and G. Isola , “The Step Further Smile Virtual Planning: Milled Versus Prototyped Mock‐Ups for the Evaluation of the Designed Smile Characteristics,” BMC Oral Health 20 (2020): 165. 32503567 10.1186/s12903-020-01145-z PMC7275593

[16] T. Joda , U. Brägger , and G. O. Gallucci , “Digital Dentistry and the Evolution of Dental Prostheses: A Scoping Review,” International Journal of Prosthodontics 32, no. 2 (2019): 201211.

[17] P. Gakis , E. Kontogiorgos , S. Zeller , and W. W. Nagy , “Effect of Firing and Fabrication Technique on the Marginal Fit of Heat‐Pressed Lithium Disilicate Veneers,” Journal of Prosthetic Dentistry 127, no. 1 (2022): 154–160, 10.1016/j.prosdent.2020.11.006. 33341255

[18] M. T. R. Corrêa , A. R. Faria , M. V. F. Melo , et al., “The Main Techniques of Flip Sculpting and Filling in Orofacial Harmonization,” Research, Society and Development 11, no. 12 (2022).

